# Extracellular Vesicles in Regenerative and Cosmetic Medicine: Safety, Clinical Effectiveness, Therapeutic Applications, and Regulatory Challenges

**DOI:** 10.3390/ijms27125541

**Published:** 2026-06-19

**Authors:** Candelaria Contreras, Amin Ariza-Donado

**Affiliations:** Escuela Dr. Amin Ariza, Barranquilla 080015, Colombia; direccion@escuelaaminariza.edu.co

**Keywords:** extracellular vesicles, small extracellular vesicles, exosomes, regenerative medicine, cosmetic dermatology, skin rejuvenation, hair restoration, wound healing, regulation

## Abstract

Extracellular vesicles (EVs), particularly small extracellular vesicles (sEVs), are lipid bilayer-delimited particles involved in intercellular communication through the transfer of proteins, lipids, and nucleic acids; many products and studies in aesthetic medicine refer to these preparations as exosomes, although endosomal origin is not always demonstrated. This review examines current evidence on the mechanisms, clinical effectiveness, safety, therapeutic applications, and regulatory challenges of EV- and sEV-based interventions, complemented by an exploratory qualitative assessment of physicians’ perceptions regarding clinical implementation. A narrative review of studies indexed in Scopus and PubMed was conducted with emphasis on skin rejuvenation, hair restoration, wound healing, pigmentation disorders, and inflammatory dermatoses, and responses from 12 aesthetic physicians in Colombia were analyzed qualitatively. Available evidence suggests that EVs/sEVs may promote extracellular matrix remodeling, angiogenesis, immunomodulation, and tissue repair, with potential benefits across several aesthetic and regenerative indications. However, the literature remains heterogeneous and limited by variability in biologic sources, isolation and administration protocols, insufficient high-quality clinical trials, and unresolved regulatory issues. Reports of adverse reactions linked to unapproved products marketed as exosome-based formulations further highlight the need for stronger oversight. EVs, particularly sEVs, often referred to as exosomes in the aesthetic literature, remain a promising therapeutic platform, but safe clinical integration requires rigorous validation, technical standardization, and robust regulatory frameworks.

## 1. Introduction

**Figure 1:**
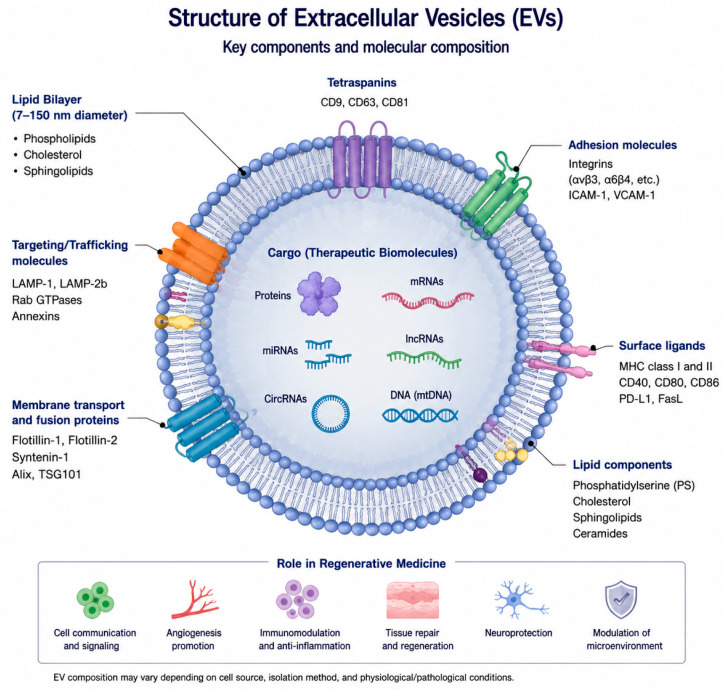
Schematic representation of extracellular vesicles (EVs), illustrating their lipid bilayer membrane enriched with phospholipids, cholesterol, sphingolipids, and ceramides, as well as characteristic surface-associated molecules such as tetraspanins, integrins, adhesion molecules, membrane trafficking proteins, and immune-related ligands. EVs encapsulate a heterogeneous bioactive cargo, including proteins, lipids, metabolites, mRNAs, miRNAs, lncRNAs, circRNAs, and DNA, which collectively mediate intercellular communication and contribute to key mechanisms involved in regenerative medicine, including angiogenesis, immunomodulation, anti-inflammatory signaling, tissue repair, neuroprotection, and microenvironmental remodeling. This schematic was created by the authors based on current extracellular vesicle characterization guidelines and published literature on EV-mediated regenerative processes.

Extracellular vesicles (EVs) are lipid bilayer-delimited particles released by multiple cell types and unable to replicate independently. Small extracellular vesicles (sEVs) are a size-based EV subpopulation, often described as less than 200 nm in diameter, but they should not be considered synonymous with exosomes unless endosomal origin is demonstrated. EVs play an important role in intercellular communication and are increasingly recognized for their translational relevance in disease diagnosis, drug delivery, and regenerative medicine (Figure 1) [1]. Each of the reports in the literature was cited respecting the tone with which the authors present the results of their studies; some use an optimistic, idealistic, and promising tone; however, as authors, we intend to make it clear that more rigor is still needed, more high-impact studies with a large number of participants, and studies with greater methodological rigor. “Extracellular vesicles, as well as adeno-associated viruses and lipid nanoparticles, have been identified among a broad range of carriers or delivery platforms. EVs, naturally secreted lipid-bilayer vesicles, have attracted great interest due to their intrinsic biocompatibility, low immunogenicity, long circulation time, and ability to cross biological barriers. These features position them as attractive vehicles that complement synthetic nanocarriers for nucleic acid therapies.” [2].

The chemical and colloidal stability of exosomes, or small extracellular vesicles, in culture media depends on multiple variables, including the organism or cell type of origin, medium composition, pH, temperature, particle concentration, incubation time, and the isolation/characterization workflow [1]. From a chemical perspective, the lipid bilayer of exosomes provides relative stability against immediate degradation and helps protect their molecular cargo, including proteins, lipids, and nucleic acids [3].

In oncology, EVs can function as noninvasive biomarkers because they transport disease-related proteins, lipids, and nucleic acids, and they have also been explored as engineered delivery systems capable of transporting therapeutic cargoes to tumor cells [4,5,6,7,8,9]. Extracellular vesicles are currently used in oncology mainly in two areas: to interrogate the tumor and to explore therapeutic strategies [1].

At present, their most advanced application is in biomarker development and liquid biopsy. The rationale is robust: these vesicles are released directly from tumor cells, circulate in biofluids such as blood and urine, and protect their molecular cargo—including RNA, DNA, and proteins—from degradation. For this reason, they are particularly attractive for diagnosis, risk stratification, and response monitoring. A 2025 review in Nature Reviews Clinical Oncology specifically describes them as an accessible source of tumor-derived information with potential applications in diagnosis, longitudinal follow-up, and clinical management [10].

There are already concrete examples of this translational trajectory. In prostate cancer, a prospective study evaluated a urinary EV-associated RNA assay, described by the authors as an exosomal RNA assay, in patients with suspected cancer prior to biopsy. The test reported a negative predictive value (NPV) of 89%, a sensitivity of 92%, and an area under the curve (AUC) of 0.67, outperforming two clinical risk calculators assessed in the same study. This does not mean that it “replaces” tissue biopsy, but it does show that the technology can already provide real clinical value in selected settings [11].

In glioblastoma, a prospective study assessed MGMT methylation in DNA associated with small extracellular vesicles and found a sensitivity of 85.7% in liquid biopsy, in addition to a longitudinal association with radiological progression in several patients. Although this was an exploratory and limited study and therefore does not alter standard clinical practice, it clearly illustrates where these approaches may be particularly useful: in tumors for which tissue is difficult to obtain or repeated biopsy-based monitoring is not feasible [12].

A second major application of EVs in oncology is their use as a functional window into cancer behavior. They not only reflect the tumor, but also actively participate in disease progression. A landmark study showed that integrins expressed on tumor-derived EVs, described in the original study as exosomes, are associated with organ-specific metastatic tropism and contribute to the formation of the pre-metastatic niche in specific organs, such as the lung and liver. This finding was important because it suggested that both the cargo and surface composition of tumor-derived EVs may be useful not only for diagnostic purposes but also for understanding and potentially anticipating patterns of metastatic dissemination [13].

In drug delivery research, EVs have attracted attention because of their biologic compatibility, their ability to cross biologic barriers, and their comparatively low immunogenicity. These properties may improve the precision and effectiveness of selected therapeutic strategies [14,15,16,17,18,19].

In drug delivery research, small extracellular vesicles (sEVs) are being investigated as biological nanocarriers. The MISEV2023 guidelines recommend the use of the terms “EVs” or “sEVs” when endosomal origin has not been rigorously demonstrated, as a substantial portion of the literature uses the term “exosomes” to describe heterogeneous or mixed vesicle preparations [1].

These vesicles function as natural delivery vehicles. Recent reviews have highlighted several advantages: they possess a lipid bilayer, protect therapeutic cargo, are capable of transporting small molecules, RNA, DNA, proteins, and other bioactive agents, and generally exhibit favorable biocompatibility compared with synthetic delivery systems. In addition, there is growing interest in their tissue penetration, their potential intrinsic tropism toward specific tissues, and their ability to traverse biological barriers [20].

They have also demonstrated considerable utility in drug delivery research for five main reasons. First, they can enhance the stability of therapeutic agents or genetic material. Second, they may improve delivery to the target tissue, either through intrinsic membrane properties or through surface engineering. Third, they provide a platform for investigating non-viral gene therapy, as they can carry transgenes, coding and non-coding RNAs, and proteins. Fourth, they are being explored for their ability to cross challenging biological barriers, such as the blood–brain barrier. Fifth, they offer a promising platform for personalized medicine by using vesicles derived from specific cell types or engineered to more effectively recognize a particular tumor or organ [21].

This utility is particularly evident in oncology. A widely cited study published in Nature reported that engineered vesicles, described by the authors as exosomes, carrying siRNA against KRAS G12D achieved greater circulation retention than liposomes in murine models, suppressed pancreatic tumors, and improved survival across several preclinical models. That work became a strong proof of concept that EV-based platforms can serve as therapeutic RNA delivery vehicles for challenging oncologic targets [22].

That concept has already progressed into early clinical evaluation. A phase I study of iExoKrasG12D in metastatic pancreatic cancer reported that the product was well tolerated, with no dose-limiting toxicities and no maximum tolerated dose reached, together with signals of disease control in some patients. Although this does not mean that it is already an established therapy, it does indicate that engineered EV-based platforms, including products described as exosome-based in the original studies, are now being evaluated as genuine clinical systems for siRNA delivery [23].

There is substantial interest in their application to gene therapy and genome editing. One review concluded that EVs can be engineered as non-viral delivery vehicles for transgenes, RNA, DNA, and functional proteins, given that gene therapy continues to face limitations with other vector platforms, particularly in terms of tissue specificity, immunogenicity, and cargo capacity [21].

Another relevant application is that they serve as an interface for studying the optimization of drug loading. The literature describes methods such as incubation, electroporation, sonication, and transfection to introduce drugs or nucleic acids into vesicles. Within this line of research, key parameters under evaluation include what can be delivered, how much can be delivered, with what degree of stability, with what intracellular release profile, and with what extent of vesicle integrity loss during the loading process [20].

Preclinical and translational research has also highlighted potential roles for EVs in wound healing and skin regeneration, osteoarthritis and cartilage repair, post-infarction cardiac repair, neurologic regeneration, kidney and liver injury, and ophthalmologic disorders [17].

Within this broader context, EVs and sEVs, commonly referred to as exosomes in the aesthetic literature, have generated substantial interest in regenerative medicine and cosmetic dermatology. The present review synthesizes available evidence on mechanisms of action, therapeutic applications, clinical effectiveness, safety concerns, and regulatory limitations, while also incorporating an exploratory qualitative perspective from aesthetic physicians regarding the requirements for safe clinical implementation.

## 2. Molecular Structure of Small Extracellular Vesicles Used in Regenerative Medicine

Small extracellular vesicles (sEVs) used in regenerative medicine are biological nanovesicles enclosed by a lipid bilayer and secreted by cells as part of their intercellular communication machinery. From a strictly terminological standpoint, the MISEV2023 guidelines recommend the use of EVs or operational terms such as sEVs when endosomal origin has not been directly demonstrated; the term exosome should be reserved for EVs from internal compartments released via multivesicular bodies. These particles are typically nanoscale in size, most commonly reported within a diameter range of 30 to 150 nm, although structurally many are classified within the broader category of small vesicles measuring less than 200 nm. When endosomal origin is demonstrated, these vesicles are generated within multivesicular bodies and released upon fusion of these compartments with the plasma membrane [1].

The molecular structure of these vesicles is primarily composed of a lipid bilayer enriched in cholesterol, sphingomyelin, ceramides, phosphatidylserine, and phosphatidylcholine. This composition confers physicochemical stability to the membrane, preserves the integrity of the intravesicular cargo, and facilitates selective interactions with target cells. Membrane-associated proteins are frequently identified on the vesicle surface, including the tetraspanins CD9, CD63, and CD81, as well as proteins involved in endosomal biogenesis and intracellular trafficking, such as ALIX and TSG101. Collectively, these components serve as EV/sEV-associated molecular markers for experimental characterization, but they should not be considered exclusive markers of exosomes; they also play active roles in cellular recognition, adhesion, internalization, and signal transduction [24].

Within their lumen, sEVs harbor a complex biological cargo composed of proteins, lipids, messenger RNA (mRNA), microRNA (miRNA), and other nucleic acids, the molecular composition of which is determined by the cell of origin and its physiological state. In aesthetic medicine, this bioactive cargo is of particular relevance because it has the capacity to modulate inflammatory responses, stimulate angiogenesis, enhance cellular proliferation, and promote extracellular matrix synthesis and remodeling. For this reason, mesenchymal stem cell-derived EVs/sEVs have attracted considerable interest as a cell-free therapeutic strategy, as they convey bioactive signals capable of promoting the repair and regeneration of skin, bone, cartilage, and other tissues [25].

From a regenerative standpoint, the therapeutic applicability of these vesicles lies not only in their nature as extracellular nanostructures, but also in the integrated organization of their molecular architecture. In this context, the vesicular membrane performs protective, stabilizing, and biological targeting functions, whereas the intravesicular cargo acts as a molecular information transfer system. Through these mechanisms, EVs/sEVs are capable of modulating the activity of recipient cells and inducing responses associated with tissue repair and regeneration without requiring the transplantation of viable cells. In parallel, platelet-rich plasma-derived EVs/sEVs have also been investigated, as their content of bioactive factors, lipids, and genetic material confers significant potential in wound healing and cutaneous regeneration processes [26].

Despite their recognized therapeutic potential, significant challenges still remain for their clinical application, including the standardization of isolation procedures, preparation purity, rigorous molecular characterization, and batch-to-batch reproducibility. Accordingly, current research is focused on precisely defining both their molecular composition and their methods of isolation prior to assigning specific therapeutic functions to them [1].

## 3. Materials and Methods

A narrative review was conducted using studies identified in Scopus, with emphasis on EV- and sEV-based applications in skin rejuvenation, hair restoration, wound healing, pigmentation disorders, inflammatory dermatoses, and related safety or regulatory issues. The search strategy included the terms “exosomes,” “extracellular vesicles,” and “small extracellular vesicles” to capture the terminology used in the aesthetic and regenerative medicine literature; however, terminology in this review follows MISEV2023 recommendations whenever possible. The objective was to provide a thematic synthesis of current evidence rather than a quantitative meta-analysis.

In addition, an exploratory qualitative assessment was performed using responses from 12 aesthetic physicians in Colombia. The responses were reviewed thematically to identify perceived barriers, educational needs, and practical considerations associated with the clinical incorporation of EV- and sEV-based interventions and products marketed as exosome-based interventions.

## 4. Mechanisms of Action of EVs/sEVs in Regenerative and Cosmetic Medicine

EVs and sEVs facilitate intercellular communication by transporting proteins, lipids, and nucleic acids capable of modulating the tissue microenvironment and promoting repair. Despite these apparent benefits, important challenges remain in isolation methods, instability of biologic sources, and the need for clinical trials to confirm long-term safety and efficacy [27,28,29].

EVs/sEVs have also been reported to regulate gene expression and induce cellular differentiation, thereby contributing to improved skin health and tissue remodeling [30]. Proposed downstream effects include extracellular matrix remodeling, angiogenesis, inflammatory modulation, and enhanced tissue repair, all of which help explain the therapeutic interest surrounding EV- and sEV-containing products, many of which are marketed or reported as exosome-based products in regenerative and aesthetic settings.

## 5. Therapeutic Applications

### 5.1. Skin Rejuvenation

EV- and sEV-based preparations, commonly described as exosomes in the aesthetic literature, have been used for skin rejuvenation with the aim of reducing wrinkles, improving skin texture and hydration, and enhancing elasticity [30,31,32]. They may stimulate extracellular matrix production and inhibit matrix metalloproteinases involved in skin aging [32].

### 5.2. Hair Restoration

Another cosmetic application of these nanoparticles is the stimulation of hair growth. EVs/sEVs may enhance follicular ce combination therapies for improved outcomes in aesthetic ll activity, modulate inflammation, stimulate dermal papilla cells, promote angiogenesis, and activate hair follicle stem cells. However, long-term studies are still needed to establish the effectiveness and safety of these treatments [27,32,33,34].

### 5.3. Wound Healing and Pigmentation Disorders

EVs/sEVs have also demonstrated usefulness in wound healing and in the management of pigmentation disorders by promoting wound closure and tissue repair [27,28,35,36].

### 5.4. Inflammatory Dermatoses

EVs/sEVs are additionally being investigated as modulators of inflammatory skin conditions, with potential applications in atopic dermatitis, psoriasis, and vitiligo [30,37].

## 6. Clinical Effectiveness, Safety, and Regulatory Challenges

Early clinical reports and small studies have described improvements in skin quality and hair-related outcomes after EV- and sEV-based interventions or products marketed as exosome-based interventions. However, the evidence base remains heterogeneous and limited by source variability, insufficient standardization, and the absence of robust long-term controlled trials.

These concerns are illustrated by case reports indicating that intradermal injection of unapproved products marketed as exosome-based formulations may lead to severe and persistent cutaneous complications. Reported adverse events, together with additional anecdotal events described in the literature, underscore the need to strengthen regulation, improve professional training, and raise public awareness in order to prevent unauthorized use of these products and protect patient safety in aesthetic practice [27,33,38].

Major challenges also remain regarding the standardization of EV/sEV production and application. Human platelet extract-based protocols have shown encouraging effects in indicators of aging and photodamage, but the available protocols still lack robust scientific support [27,32,35].

Products marketed as exosome-based treatments have not yet been fully approved by regulatory agencies such as the U.S. Food and Drug Administration [39], and additional clinical validation is required to establish their safety and efficacy [29,32,33]. Ongoing research is essential to overcome current limitations and to more clearly define the scope of EV/sEV applications in cosmetic dermatology [27,35,37]. Future applications may include engineered EVs/sEVs with enhanced targeting capabilities and combination therapies integrating EVs/sEVs with established dermatologic procedures [37].

## 7. Combination Protocols with Microneedling and Energy-Based Devices

EV/sEV preparations, including products described as exosome-based, have been used in combination with procedures such as microneedling, radiofrequency, and other energy-based treatments. These protocols may enhance skin rejuvenation, accelerate recovery, and support longer-lasting results, although standardization and regulatory approval remain limited (1).

A retrospective study involving 40 patients used a preparation described by the authors as exosomes at a concentration of 50 mcg/mL applied with microneedling every two weeks for four sessions. The authors reported high patient satisfaction and clinically relevant skin improvement [28].

Another protocol combined superficial microneedling (0.3 mm) with topical EV/sEV preparations described as exosomes and reported sustained improvements in pore size, erythema, and pigmentation over 21 months, suggesting possible long-term biologic restructuring [40].

In a comparative trial, adipose-derived stem cell EVs/sEVs described by the authors as exosomes were compared with platelet-rich plasma on opposite sides of the face during three microneedling sessions with additional radiofrequency. Both interventions showed similar improvements in wrinkles, dyschromia, skin texture, collagen, and glycosaminoglycan content [41].

Current evidence also highlights the combination of EVs/sEVs, frequently described as exosomes in the aesthetic literature, with lasers and related energy-based treatments to optimize neovascularization, extracellular matrix remodeling, post-procedural recovery, and overall skin quality [42,43].

## 8. Cosmetic vs. Therapeutic Exosomes: Regulatory Differences

The regulatory and compliance distinctions between cosmetic/aesthetic exosome-based products and therapeutic exosome-based products are primarily determined by the product’s intended use, route of administration, composition, and promotional claims. When an exosome-containing product is marketed exclusively for cosmetic purposes, such as beautifying, moisturizing, protecting, or improving the appearance of the skin, it may fall within the cosmetic regulatory framework; in this context, compliance generally focuses on safety substantiation, truthful and non-misleading labeling, good manufacturing practices, facility and product registration or listing where applicable, and post-market adverse event reporting [44,45]. In the United States, cosmetic products and ingredients generally do not require FDA premarket approval, except for color additives, although MoCRA has expanded FDA oversight by introducing additional obligations related to safety substantiation, facility registration, product listing, and serious adverse event reporting. In the European Union, cosmetic products must comply with Regulation (EC) No 1223/2009, which requires the designation of a responsible person, a cosmetic product safety assessment, a Product Information File, compliance with good manufacturing practice, and electronic notification prior to market placement [46]. Conversely, when an exosome-based product is intended or promoted to diagnose, treat, cure, mitigate, or prevent disease, or to exert biological effects such as tissue regeneration, wound healing, immune modulation, or anti-inflammatory activity, it is generally regulated as a drug, biological product, or advanced therapy medicinal product, depending on the jurisdiction. Such classification triggers a substantially more stringent regulatory pathway, including preclinical characterization, quality and manufacturing controls, authorization to conduct clinical investigations, generation of clinical evidence demonstrating safety and efficacy, and premarket authorization through mechanisms such as an IND followed by a BLA/NDA in the United States, or a marketing authorization pathway for advanced therapy medicinal products in the European Union [47]. The FDA has specifically stated that there are currently no FDA-approved exosome products and has issued warning letters indicating that exosome products promoted with regenerative, wound-healing, anti-inflammatory, or tissue-repair claims may be considered unapproved drugs and unlicensed biological products [48]. Additionally, in the EU cosmetic context, cells, tissues, or products of human origin are listed among prohibited substances under Annex II of the Cosmetics Regulation, which is a critical consideration for exosome-containing cosmetic formulations derived from human biological materials [46].

## 9. Exploratory Qualitative Assessment of Aesthetic Physicians’ Perspectives

As a complement to the literature review, and considering the still limited number of scientific articles addressing the use of EVs/sEVs or products marketed as exosome-based in aesthetic treatments, an exploratory assessment was conducted in a community of 12 aesthetic physicians in Colombia.

Several recurrent themes emerged from the responses. Participants emphasized the need for stronger scientific support and higher-level evidence, interest in structured and formal training, continuous updates on new findings, clearer protocols and indication-based guidance, interest in techniques that may improve outcomes, and broader comprehensive training in the clinical use of EVs/sEVs and products marketed as exosomes.

Overall, the confidence in the use of EVs/sEVs among the surveyed aesthetic physicians seemed to depend on three fundamental elements: solid scientific evidence, practical and applied training, and clear and structured clinical protocols.

## 10. Conclusions

EVs, particularly sEVs, often referred to as exosomes in the aesthetic literature, are emerging as a cell-free therapeutic alternative of considerable interest in regenerative and cosmetic medicine because of their capacity to intervene in key processes such as angiogenesis, immunomodulation, extracellular matrix remodeling, and tissue repair. The reviewed evidence suggests potential benefit in indications such as skin rejuvenation, hair restoration, wound healing, and certain inflammatory and pigmentary dermatoses. However, these findings should be interpreted with caution because the field remains characterized by marked heterogeneity in biologic sources, isolation methods, product characterization, routes of administration, and clinical outcomes.

The absence of high-quality controlled clinical trials, the limited standardization of protocols, and the persistence of regulatory gaps currently restrict the consolidation of EVs/sEVs as a fully validated intervention for aesthetic practice. This is compounded by concerns regarding adverse events associated with unapproved formulations, reinforcing the need for stricter surveillance and robust criteria for clinical use. In parallel, the qualitative exploration conducted in this study indicates that adoption by aesthetic physicians depends not only on promising outcomes but also on solid scientific evidence, practical training, and clearly defined clinical protocols.

Accordingly, although EVs, particularly sEVs, often referred to as exosomes in the aesthetic literature, represent a platform with notable translational potential, their responsible incorporation into aesthetic medicine requires rigorous clinical validation, technical standardization, regulatory oversight, and evidence-based professional training. Only under these conditions will it be possible to translate their biologic promise into reproducible, safe, and ethically sustainable clinical benefits.

**Figure 2:**
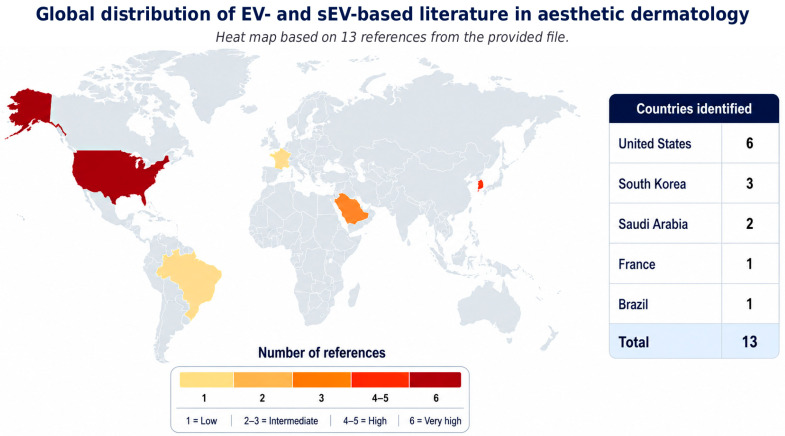
World heat map of countries represented by the selected research references on EV- and sEV-based or exosome-described applications in regenerative/cosmetic medicine. Country counts represented in the heat map: United States (6), South Korea (3), Saudi Arabia (2), France (1), Brazil (1). The data in Figure 2 are derived from Table 1.

Taken together, Table 1 and Figure 2 present the countries actively studying EV- and sEV-based or exosome-described applications in regenerative medicine.

## Figures and Tables

**Table 1:** References and countries of studies reporting EV- and sEV-based or exosome-described cosmetic/aesthetic applications.

Ref.	References	Country
[27]	Haykal D, Wyles S, Garibyan L, Cartier H, Gold M. Exosomes in cosmetic dermatology: A review of benefits and challenges. J Drugs Dermatol. 2025;24(1):12–18. doi:10.36849/JDD.8872.	France
[28]	Cho B, Bae KT, Wan J, Yi KH. Effects of exosome-containing skin booster and microneedling treatment on facial aging: A retrospective analysis of 40 cases. Plast Aesthet Nurs. 2026;46(1):25–29. doi:10.1097/PSN.0000000000000631.	South Korea
[29]	Al Ameer M, Alnajim A, Al Ameer A, Alsalman Z, Al Ameer G, Alnajim S et al. Exosomes and hair regeneration: A systematic review of clinical evidence across alopecia types and exosome sources. Clin Cosmet Investig Dermatol. 2025;18:2215–2227. doi:10.2147/CCID.S543451.	Saudi Arabia
[30]	Queen D, Avram MR. Exosomes for treating hair loss: A review of clinical studies. Dermatol Surg. 2025;51(4):409–415. doi:10.1097/DSS.0000000000004480.	United States
[32]	Proffer SL, Paradise CR, DeGrazia E, Halaas Y, Durairaj KK, Somenek M et al. Efficacy and tolerability of topical platelet exosomes for skin rejuvenation: Six-week results. Aesthet Surg J. 2022;42(10):1185–1193. doi:10.1093/asj/sjac149.	United States
[33]	Park KY. Adverse reactions following intradermal injection of exosome-based formulations: A case series. J Cosmet Dermatol. 2025;24(10):e70520. doi:10.1111/jocd.70520.	South Korea
[35]	AlBargawi S. Necrosis following dermal injection of lyophilized exosomes: A case report. J Cosmet Dermatol. 2025;24(8):e70387. doi:10.1111/jocd.70387.	Saudi Arabia
[37]	Dal’Forno-Dini T, Birck MS, Rocha M, Bagatin E. Exploring the reality of exosomes in dermatology. An Bras Dermatol. 2025;100(1):121–130. doi:10.1016/j.abd.2024.09.002.	Brazil
[38]	Shah M, Dukharan V, Broughton L, Stegura C, Schur N, Samman L et al. Exosomes for aesthetic dermatology: A comprehensive literature review and update. J Cosmet Dermatol. 2025;24(1):e16766. doi:10.1111/jocd.16766.	United States
[40]	Lee YS. Regenerative skin remodeling through exosome-based therapy: A case study demonstrating 21-month sustained outcomes in pore size, erythema, and hyperpigmentation. Dermatol Ther (Heidelb). 2025;15(10):3055–3064. doi:10.1007/s13555-025-01501-3.	South Korea
[41]	Estupiñan B, Ly K, Goldberg DJ. Adipose mesenchymal stem cell-derived exosomes versus platelet-rich plasma treatment for photoaged facial skin: An investigator-blinded, split-face, non-inferiority trial. J Cosmet Dermatol. 2025;24(5):e70208. doi:10.1111/jocd.70208.	United States
[42]	Peredo M, Shivananjappa S. Topical human mesenchymal stem cell-derived exosomes for acceleration of wound healing following tissue trauma and aesthetic procedures: A case series. J Drugs Dermatol. 2024;23(4):281–284. doi:10.36849/JDD.C7395.	United States
[43]	Duncan DI, Tiryaki T, Suwanchinda A, Chernoff G, Cohen S. Exosomes in aesthetic medicine: An overview. Aesthet Surg J. 2026;46(Suppl 1):S1-S12. doi:10.1093/asj/sjaf259.	United States

## Data Availability

No new data were created or analyzed in this study. Data sharing is not applicable to this article.
